# Association of exposure to indoor molds and dampness with allergic diseases at water-damaged dwellings in Korea

**DOI:** 10.1038/s41598-023-50226-w

**Published:** 2024-01-02

**Authors:** Seokwon Lee, Seung-Hun Ryu, Woo Jun Sul, Seunghyun Kim, Dohyeong Kim, SungChul Seo

**Affiliations:** 1 Environmental Health Research Department, National Institute of Environmental Research, Incheon, 22689 Republic of Korea; 2 Department of Environmental Health Sciences, Graduate School of Public Health, Seoul National University, Seoul, 08826 Republic of Korea; 3 Department of Systems Biotechnology, College of Biotechnology and Natural Resources, Chung-Ang University, Anseong, Gyeonggi-do, 17546 Republic of Korea; 4 Allergy Immunology Center, College of Medicine, Korea University, Seoul, 02841 Republic of Korea; 5 School of Economic, Political and Policy Sciences, University of Texas at Dallas, Richardson, TX 75080-3021 USA; 6 Department of Nano, Chemical and Biological Engineering, College of Engineering, Seokyeong University, Seoul, 02173 Republic of Korea

**Keywords:** Environmental sciences, Public health

## Abstract

This study aims to characterize levels of molds, bacteria, and environmental pollutants, identify the associations between indoor mold and dampness exposures and childhood allergic diseases, including asthma, allergic rhinitis, atopic dermatitis, using three different exposure assessment tools. A total of 50 children with their parents who registered in Seoul and Gyeonggi-do in Korea participated in this study. We collated the information on demographic and housing characteristics, environmental conditions, and lifestyle factors using the Korean version of the International Study of Asthma and Allergies in Childhood questionnaire. We also collected environmental monitoring samples of airborne molds and bacteria, total volatile organic compounds, formaldehyde, and particulate matter less than 10 µm. We evaluated and determined water damage, hidden dampness, and mold growth in dwellings using an infrared (IR) thermal camera and field inspection. Univariate and multivariate regression analyses were performed to evaluate the associations between prevalent allergic diseases and exposure to indoor mold and dampness. Indoor mold and bacterial levels were related to the presence of water damage in dwellings, and the mean levels of indoor molds (93.4 ± 73.5 CFU/m^3^) and bacteria (221.5 ± 124.2 CFU/m^3^) in water-damaged homes were significantly higher than those for molds (82.0 ± 58.7 CFU/m^3^) and for bacteria (152.7 ± 82.1 CFU/m^3^) in non-damaged dwellings (*p* < 0.05). The crude odds ratios (ORs) of atopic dermatitis were associated with < 6th floor (OR = 3.80), and higher indoor mold (OR = 6.42) and bacterial levels (OR = 6.00). The crude ORs of allergic diseases, defined as a group of cases who ever suffered from two out of three allergic diseases, e.g., asthma and allergic rhinitis, and allergic rhinitis were also increased by 3.8 and 9.3 times as large, respectively, with water damage (+) determined by IR camera (*p* < 0.05). The adjusted OR of allergic rhinitis was significantly elevated by 10.4 times in the water-damaged dwellings after adjusting age, sex, and secondhand smoke. Therefore, a longitudinal study is needed to characterize dominant mold species using DNA/RNA-based sequencing techniques and identify a causal relationship between mold exposure and allergic diseases in the future.

## Introduction

There are various types of microorganisms in residential housing, and mold and bacteria can progressively grow as temperature and humidity increase in the indoor environment^1–5^. Water damage caused by flooding and leakages, even if invisible to the residents, also makes them grow rapidly, spreading over larger areas in the walls and ceilings of the residential house buildings; thus, the risk of inhalation exposure to various microorganisms for residents can also be increased in indoor air^6–8^. In this regard, it is highly necessary to conduct a study evaluating biological risk factors, including molds and bacteria that could grow due to flooding and leakages in indoor dwellings during heavy rainfalls, and identify the association between the exposure risk and health effects among residents.

In Korea, excessive moisture (dampness) and water damage in the indoor environment of residential complex buildings can occur due to frequent heavy rainfalls and typhoons in summer and autumn seasons^9–11^, and the exposure to visible mold or dampness was significantly associated with increased risks of atopic dermatitis (AD)^12,13^ and allergic rhinitis^14,15^ among Korean children. In most previous studies, conventional approaches of exposure assessment for indoor moisture (humidity) and microorganisms were generally used to collect (measure) air samples on site and quantitatively evaluate them by incubating, characterizing^6,16–18^, and analyzing in the laboratory or conducting questionnaire-based surveys and interviews with individual residents to collect detailed qualitative information on various environmental factors that may affect moisture (dampness) and inhalation exposures to indoor molds and bacteria^14,19–21^. However, these approaches for exposure assessment have some limitations, including low reliability and reproducibility, uncertainty, and misclassification bias, in the results.

In several recent studies, a new technique, infrared (IR) thermographic imaging, was used to detect the difference in temperature on the surface of building walls and ceilings for determining the origin of water damage caused by flooding and leakages as well as high levels of moisture (dampness) and to evaluate potential mold exposure by regarding the darker images with lower temperature as the surfaces or areas damaged by water or moisture from the outdoor environment^22–26^. In particular, a cross-sectional study conducted by Seo et al*.* reported that higher levels of airborne mold exposure were significantly associated with water-damaged homes, and the severity of AD for children living in water-damaged homes with visible molds and water stains assessed by IR camera was significantly increased. The authors, including pediatric allergists, evaluated SCORing Atopic Dermatitis (SCORAD) scores to classify the severity of AD into mild, moderate, and severe groups, and they found that moisture problems at homes are positively associated with the severity of AD^27^.

However, the authors^27^ noted that further study is still needed to identify whether the high levels of airborne molds and bacteria resulted from water damage and moisture problems at indoor residential dwellings and whether the origin of indoor mold and bacterial growth came from outdoor environments. Furthermore, they also suggested which method of mold exposure assessment would be a proper, efficient, and reliable one among various methods, such as questionnaire-based surveys, environmental monitoring sampling with culture-based analysis, on-site field inspection (visual observation), and IR thermal imaging techniques, for the identification of the associations between prevalent allergic diseases, including asthma and allergic rhinitis, and exposure to environmental risk factors. However, there has been little study investigating exposure to indoor mold and water damage determined using the IR cameras and identifying the association between indoor mold exposure and childhood allergic diseases. Therefore, this study aims to characterize the levels of inhalation exposure to airborne molds, bacteria, and environmental pollutants in the indoor and outdoor environments using the ISAAC questionnaire survey, environmental sampling measurement, and IR thermal imaging technique and to identify the associations between the inhalation exposure to biological and environmental pollutants and prevalent allergic diseases among children in Korea.

## Methods

### Study participants and questionnaire surveys

**Figure 1:**
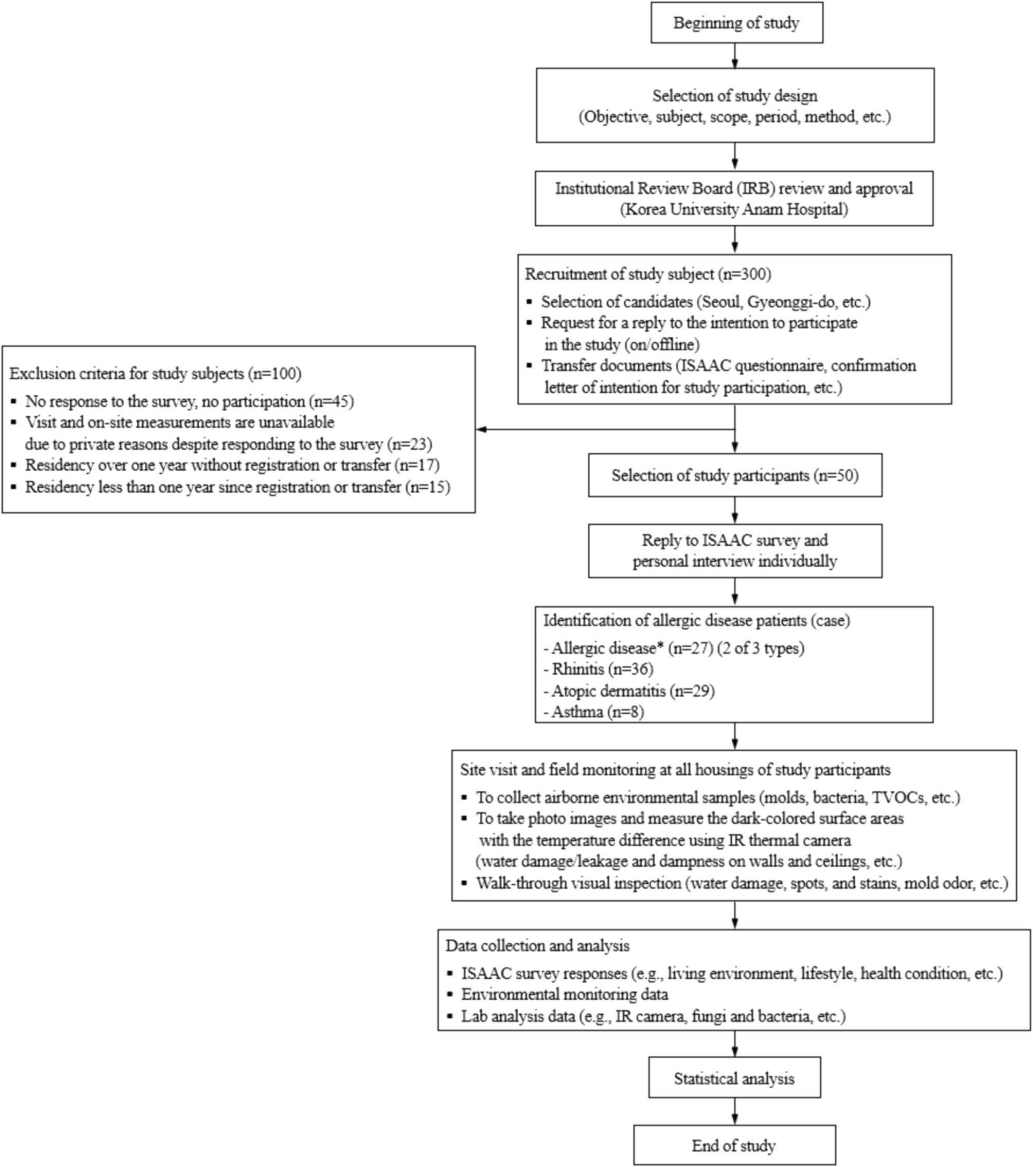
A schematic diagram of the study design, key methodology, and process.

Prior to the beginning of the present study, we explained the study background, rationale, objectives, and detailed plans to all candidate study participants and received written application documents from households. A total of 50 children with legal parents who voluntarily agreed to perform environmental sampling measurements and questionnaire surveys, including on-site visual inspection for evaluation of the presence of water damage, dampness, and visible molds and registered as residents in Seoul city and Gyeonggi Province in Republic of Korea from September to November 2019, were randomly recruited and participated in this study. However, preschool or early elementary school-aged children can neither participate alone nor directly respond to the environmental monitoring measurements and questionnaire surveys in the real-world situation. Thus, their legal guardians (or parents) participated in the present study. The overall study design, key methodology, and process is drawn as Fig. 1.

All the parents responded to a standardized Korean version of the International Study of Asthma and Allergies in Childhood (ISAAC) questionnaire^21,28–30^ and provided detailed information on current health status and past medical history for their children and whether they were ever diagnosed with asthma, allergic rhinitis, or AD. Cases or patient households were defined as children who had been “ever diagnosed” with allergic diseases (asthma, allergic rhinitis, or AD) in the past by professional pediatric allergists or who visited hospitals and had clinical treatment for their allergic diseases and related symptoms “within the last 12 months to date”. We also collected detailed information on the characteristics of residential housing and lifestyle factors, which might affect the levels of airborne molds, bacteria, and environmental pollutants in the indoor environment, via questionnaire surveys.

More specifically, the characteristics of residential housing included via the survey were asked to all households for type of housing (Single/multi-household house vs. Apartment complex building), building age (< 10 vs. ≥ 10 years), floor (< 6th vs. ≥ 6th), direction of housing (South vs. East/West for the direction of main living room), net square meter area of housing (< 85 m^2^ vs. ≥ 85 m^2^), and duration of residency (< 3 vs. ≥ 3 years). Furthermore, information on lifestyle factors, such as secondhand smoke (Yes vs. No), frequency of natural ventilation (Everyday vs. Less than 2–3 times per week) and housing cleaning (Everyday vs. Less than 2–3 times per week), and pets (Yes vs. No), was also collated from the questionnaire survey. The information on the presence of water damage, leakage, dampness, or visible mold growth was also collected from the ISAAC questionnaire survey. We asked whether anyone of family (household) members has ever observed (or noticed) water damage, damp stains, leakage, or visible mold growth on the floor, walls, or ceiling of their rooms or house within the last 12 months or ever (Yes vs. No).

This study was conducted in accordance with the Declaration of Helsinki, and all methods were carried out in accordance with relevant guidelines and regulations. Written informed consent was obtained from all study participants, and it was explained that all collected data and personal information would not be shared or disclosed outside this study. This study was reviewed and approved by an institutional ethics review board (IRB) of the Korea University Anam Hospital (IRB approval no. ED12221).

### Environmental sampling and analysis and exposure assessment

We visited a total of 50 households and collected air samples of airborne microorganisms (molds and bacteria) from September to November in 2019 and established the fundamental principle of indoor environmental measurement to collect air samples at locations (rooms) where the study participants use the most frequently or spend the longest period of time. The measurement points were located at the center of the bedroom or living room and within 10 m from outside the house buildings, and air samples were collected at a height of 1.2–1.5 m from the floor ground considering potential redispersion.

Airborne mold and bacteria samples were collected using an air sampler with a flow rate of 180 L/min per head, DUO Surface Air System (SAS) Super 360 (VWR International, LLC., Milan, Italy), and another one, Andersen single-stage sampler (KAS-110) with a flow rate of 28.3 L/min (KEMIK Cooperation, Seongnam, Republic of Korea). Malt extract agar medium supplemented with streptomycin sulfate 40 mg/L (Difco Laboratories, Detroit, MI, USA) was used for molds, and tryptic soy agar with an antifungal agent, cycloheximide 0.5 mg/mL (Sigma‒Aldrich, St. Louis, MO, USA), was used for bacterial sampling. To avoid cross-contamination, the lead of the samplers was disinfected and cleaned with 70% ethanol before and after air sampling. After wrapping with Parafilm® (American National Can company, Chicago, IL, USA), all the collected samples were stored in insulated containers with frozen ice packs at 4 °C and transported to an accredited analytical laboratory within 24 h.

The collected molds and bacteria were incubated at 25 °C for over 96 h and 35 °C for over 48 h, respectively, and the number of colonies was counted and corrected using the Anderson sampler positive hole conversion table^31,32^. The final concentrations of airborne microorganisms were converted and expressed as colony forming units per cubic meter (CFU/m^3^). For quality control purposes, approximately 10% of the total samples were also collected as blank samples, and several environmental factors, including temperature and relative humidity, were also measured in the indoor and outdoor environments.

Both total volatile organic compounds (TVOCs) and formaldehyde (HCHO) samples were collected from the living room and children’s bedrooms of each residence. The TVOC samples were collected using multiple Supelco Tenax TA thermal desorption tubes (Sigma‒Aldrich, St. Louis, MO, USA) with mini pumps (MP-Σ30KN II; Sibata Scientific Technology Ltd., Soka, Saitama, Japan) at a constant flow rate of 0.1 L/min for 30 min, and the samples were desorbed by a thermal desorber (Unity/Ultra TD; Markes International Ltd., Llantrisant, UK) and then quantified by a gas chromatograph (Model 6890; Agilent Technologies, Inc., Santa Clara, CA, USA) coupled to a mass selective detector (Model 5975B; Agilent Technologies, Inc., Santa Clara, CA, USA).

On the other hand, the HCHO samples were also collected using silica gel cartridges coated with 2,4-dinitrophenylhydrazine (DNPH) (SKC Inc., Eighty-Four, PA, USA) with ozone scrubbers (Waters Corp., Milford, MA, USA) on mini pumps (MP-Σ30KN II; Sibata Scientific Technology Ltd., Soka, Saitama, Japan). All the collected samples were immediately sealed and stored in insulated containers with frozen ice packs and transported to the analytical laboratory in the frozen state. Finally, the HCHO samples were analyzed using a high-performance liquid chromatographic (HPLC) system with an ultraviolet‒visible (UV‒VIS) spectrophotometry detector (Series 200; Perkin Elmer Inc., Waltham, MA, USA) at a wavelength of 365 nm. Similar to airborne mold and bacterial samples, blank samples of HCHO collected using the 2,4-DNPH tubes were also provided to the analytical laboratory for quality control purposes, and the results of blank samples were used to adjust for the concentrations of HCHO samples. Indoor and outdoor particulate matter less than 10 µm (PM_10_) was also measured using a portable optical particle counter, a portable Grimm Aerosol Spectrometer (Model 1.108, Grimm Aerosol Technik GmbH, Ainring, Germany). Two monitoring devices were installed to collect particle mass concentrations at each residential housing for a week, and another monitor was also installed and measured PM_10_ levels outside the housing.

### Evaluation of water damage using IR thermal camera

**Figure 2:**
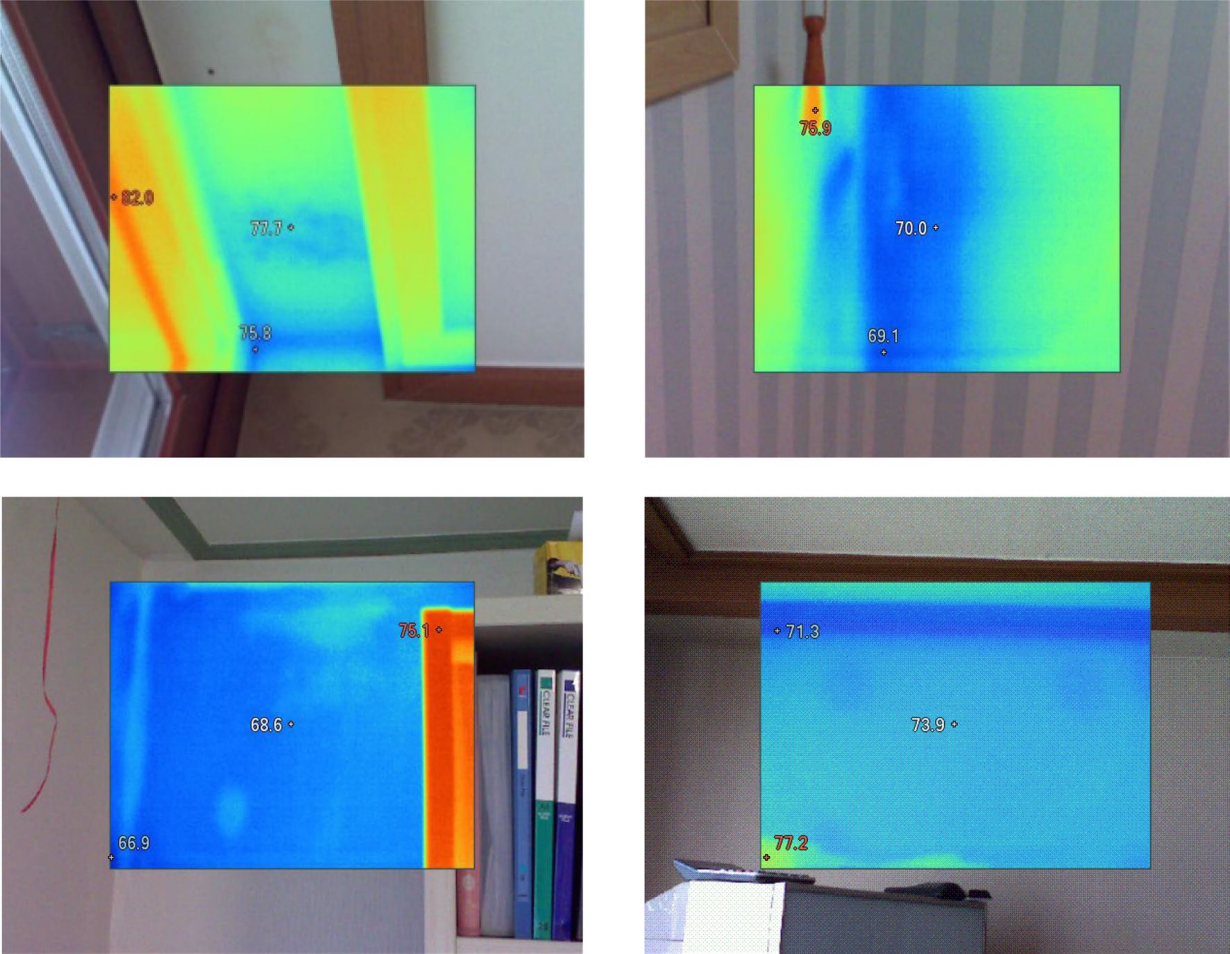
Examples of photographs with water damages and hidden dampness (dark blue colored areas) taken using an IR thermal camera at multiple locations of residential dwellings in this study.

The authors of the present study performed walk-through monitoring using an infrared (IR) thermal imaging camera and field inspection (visual observation) to determine water damage and leakage (stains) and mold growth in residential housing from September to November 2019. First, we investigated all surface areas of 50 residential dwellings and then took photographs (colored images) as surrogates of the presence of water damage and hidden dampness on the suspected areas in the walls and ceilings of dwellings using an IR camera, FLK-Ti29 60 Hz industrial-commercial thermal imager (Fluke Corporation, Everett, WA, USA). Second, on-site investigation via visual observation was also performed to identify visible molds, water leakage and stains, and moldy odor by well-trained field inspectors. After finding the existence of dampness (moisture), visible mold growth, and water leakage and stains, we double checked if the temperature (color) difference between the water-damaged and its surrounding areas from the photos previously taken was higher than 5 °C using the IR camera (Fig. 2). Similar to a previous study^27^, we used publicly available photography imaging processing software, ImageJ (https://imagej.nih.gov/ij/), to analyze two-dimensional (2D) photo images taken from the water-damaged areas. Thirdly, the presence of water damage and leakages was defined as each or all combined surface of damaged area (i.e., dark blue colored) with ≥ 0.2 m^2^ on the walls and ceilings of bedroom or living room, according to criteria by Meklin et al.^33^ Finally, the water-damaged dwellings were defined when calculating total water-damaged surface area was ≥ 0.2 m^2^ in both bed and living rooms at each home (a continuous variable). However, we defined as non-damaged dwellings (1) if the water-damaged surface areas were observed not in children’s bedroom but in living room or other rooms, (2) if all combined areas with water damage, hidden dampness, and visible mold growth were calculated less than 0.2 m^2^ in children’s bedroom and living room of house.

### Statistical analysis

In this study, all indoor and outdoor measurements of airborne molds and bacteria, TVOCs, HCHO, HDMs at water-damaged and undamaged residential dwellings, and PM_10_ were shown to be log-normally distributed after performing the Shapiro Wilk test. General characteristics of the study participants were described by the number of subjects with percentages (%), and arithmetic and geometric means (Ams and GMs) and standard deviations (SDs) were also summarized to describe the center and spread of collected exposure datasets. The significant differences between the indoor and outdoor concentration levels of airborne mold, bacterial, TVOC, HCHO, PM_10_, and HDM exposure were calculated by paired t tests.

The levels of airborne mold and bacterial exposures were statistically compared between the water-damaged and undamaged residential dwellings determined by IR thermal images and questionnaire surveys, including visual inspections on site. Simple correlation analysis was performed to calculate Pearson correlation coefficients between the presence of water damage determined by IR camera and all indoor and outdoor measurements. Multiple regression analysis with dummy variables was also performed to identify the associations between indoor mold and bacterial concentration levels (natural log-transformed) as dependent variables and various environmental and lifestyle factors (e.g., the presence of water damage and mold growth, building age, type and direction of housing, floor, secondhand smoke, frequency of natural ventilation, frequency of cleaning, and pet) as independent variables (dummy). Multicollinearity for independent variables was checked by calculating the variation inflation factor (VIF).

Univariate logistic regression analyses were performed to calculate the unadjusted odds ratios (ORs) and identify the associations between the prevalent allergic diseases (asthma, allergic rhinitis, and AD) and the levels of exposure to mold, bacteria, TVOCs, HCHO, and PM_10_ with various environmental factors. The adjusted ORs of childhood allergic diseases, allergic rhinitis, and atopic dermatitis were also estimated to identify the associations with the presence of water damage and hidden dampness determined using an IR camera and ISAAC questionnaire survey as well as high levels of indoor mold exposures, and compared by each method of exposure assessment (IR thermal camera, questionnaire survey, or environmental sampling technique) after adjusting for age, sex, and secondhand smoke in the multivariate logistic regression model. All statistical analyses were performed using STATA software version 16.1 (StataCorp LP, College Station, TX, USA) and R statistical software version 4.2.2 (R Core Team, 2022) with Rstudio version 2023.03.1 + 446 (Rstudio Inc., Boston, MA, USA), and a *p value* less than 0.05 was considered statistically significant.

## Results

Table 1 shows the general characteristics of the study participants. The participants consisted of a total of 50 children (families) in households (31 male, 19 female), and the mean age was 9.0 ± 3.1 years old, ranging from 2 to 14 years old. The major characteristics of residential housing were apartment complex buildings (n = 38, 76%), southern direction (n = 43, 86%), ≥ 10 building age (n = 35, 70%), ≥ 3 years duration of residency (n = 30, 60%), < 85 m^2^ net square meter area (n = 39, 78%), and < 6th floor (n = 26, 52%). Lifestyle factors were shown to be no secondhand smoke (n = 27, 54%), natural ventilation every day (n = 45, 90%), house cleaning every day (n = 33, 66%), and no pet (n = 39, 78%). In Table 2, the study participants responded to “Yes” for the presence of water damage, leakage, or visible mold growth at dwellings (n = 34, 68%) in the ISAAC questionnaire survey, but water damage and hidden dampness determined by the IR thermal imaging camera and field inspection was “Positive (≥ 0.2 m^2^)” (n = 16, 32%).

**Table 1:** General characteristics of the study participants who responded to the questionnaire surveys.

Characteristics	Water-damaged (n = 16)		No water-damaged (n = 34)		*p* value
	n	%	n	%	
Age					
< 10 years-old	8	50.0	20	58.8	0.56
≥ 10 years-old	8	50.0	14	41.2	
Sex					
Male	13	81.2	18	52.9	0.06
Female	3	18.8	16	47.1	
Income*					
< 2292	4	25.0	12	35.3	0.59
2292–3820	5	31.3	12	35.3	
≥ 3820	7	43.7	10	29.4	
Type of housing					
Apartment complex building	14	87.5	24	70.6	0.19
Single or multi-household house	2	12.5	10	29.4	
Building age					
< 10 years	6	37.5	9	26.5	0.43
≥ 10 years	10	62.5	25	73.5	
Duration of residency					
< 3 years	6	37.5	14	41.2	0.80
≥ 3 years	10	62.5	20	58.8	
Net square meter area**					
< 85 m^2^	15	93.8	24	70.6	0.06
≥ 85 m^2^	1	6.2	10	29.4	
Direction of housing					
South	14	87.5	29	85.3	0.83
East or West	2	12.5	5	14.7	
Floor					
< 6th	8	50.0	18	52.9	0.85
≥ 6th	8	50.0	16	47.1	
Secondhand smoke					
Yes	7	43.8	16	47.1	0.83
No	9	56.2	18	52.9	
Frequency of natural ventilation					
Everyday	14	87.5	31	91.2	0.69
2–3 times per week	2	12.5	3	8.8	
Frequency of cleaning					
Everyday	11	68.8	22	64.7	0.78
2–3 times per week	5	31.2	12	35.3	
Pet					
Yes	4	25.0	7	20.6	0.72
No	12	75.0	27	79.4	

Water-damaged dwellings are defined when all combined suface areas of dark blue colored determined using IR camera with ≥ 0.2 m^2^ on the walls and ceilings of bed and living rooms, and no water-damaged ones were calculated less than 0.2 m^2^ at each home.

*Unit: US dollar per month ($1 = 1308.9 Korean won).

**Actual occupation area of residence.

**Table 2:** Determination of the presence of water damage, hidden dampness, and visible mold growth using ISAAC questionnaire survey and IR thermal imaging camera.

Characteristics		n	%
ISAAC questionnaire survey			
Water damage, leakage, or mold growth*	Positive (Yes)	34	68.0
	Negative (No)	16	32.0
IR thermal imaging camera			
Water damage and hidden dampness	Positive (≥ 0.2 m^2^)	16	32.0
	Negative (< 0.2 m^2^)	34	68.0

*Definition: “Yes”—Any of water damage, leakage, dampness, or visible mold growth was observed; “No”—None of water damage or visible mold growth was observed in their dwellings.

The levels of airborne molds, bacteria, TVOC, HCHO, and PM_10_ measured in the indoor and outdoor environments are summarized in Table 3. The indoor mean levels of airborne mold and PM_10_ were GM 66.9 CFU/m^3^ and GM 25.3 μg/m^3^, which were significantly lower than the outdoor means (GM 95.9 CFU/m^3^ for molds and GM 32.3 μg/m^3^ for PM_10_), respectively (*p* < 0.05). In the indoor environment, the mean level of bacterial exposure in the living room (GM 184.4 CFU/m^3^) was significantly higher than that in the bedroom (GM 135.3 CFU/m^3^), whereas the mean HCHO in the bedroom (GM 52.2 μg/m^3^) was significantly higher than that in the living room (GM 41.3 μg/m^3^) (*p* < 0.05). However, the mean levels of airborne molds and TVOCs between bedroom and living room were not significantly different. Both temperature and relative humidity were significantly different between indoor and outdoor environments (*p* < 0.05), but no difference was observed between bed and living rooms. The mean levels of temperature and relative humidity were GM 23.3 °C and GM 46.6% in indoor dwellings as well as GM 15.2 °C and GM 63.3% in the outdoor environment, respectively.

**Table 3:** Summary statistics for comparison of the levels of molds, bacteria, TVOC, HCHO, PM_10_, temperature and relative humidity in water-damaged dwellings and outdoor environment.

Exposure parameter	Location	n	AM	SD	Median	Min	Max	*p* value
Mold (CFU/m^3^)								
Indoor	Living room	50	91.3	81.9	66.7	16.7	383.3	0.61
	Bedroom	50	80.0	55.4	61.5	14.6	227.1	
	Average	50	85.6	65.2	66.7	15.7	279.2	** < 0.05**
Outdoor		50	120.3	100.6	95.8	20.0	672.9	
Bacteria (CFU/m^3^)								
Indoor	Living room	50	221.6	135.1	183.3	45.8	583.3	** < 0.05**
	Bedroom	50	177.4	127.7	145.9	8.3	595.8	
	Average	50	199.5	118.2	150.0	33.3	495.8	0.35
Outdoor		50	190.8	140.6	145.8	33.3	575.0	
Total volatile organic compound (TVOC) (µg/m^3^)								
Indoor	Living room	50	350.1	238.2	313.6	66.2	937.3	0.56
	Bedroom	50	406.8	286.8	366.1	21.6	1448.2	
	Average	50	378.4	235.5	384.4	44.6	872.9	–
Formaldehyde (HCHO) (µg/m^3^)								
Indoor	Living room	50	46.2	19.5	47.7	3.6	108.5	** < 0.05**
	Bedroom	50	59.4	29.5	55.9	8.4	154.4	
	Average	50	52.8	23.7	54.1	6.0	131.4	–
Particulate matter (PM_10_) (µg/m^3^)								
Indoor	Living room	50	28.8	11.9	24.6	12.3	57.6	0.20
	Bedroom	50	26.0	11.1	24.4	11.5	58.1	
	Average	50	27.4	10.9	25.5	11.9	52.4	** < 0.05**
Outdoor		50	34.0	11.4	32.6	14.8	75.3	
Temperature								
Indoor	Living room	50	23.3	3.4	24.2	14.0	30.0	0.15
	Bedroom	50	23.6	2.7	24.6	17.0	28.0	
	Average	50	23.5	3.0	24.4	15.2	29.0	** < 0.05**
Outdoor		42	16.1	4.9	15.1	5.3	23.0	
Relative humidity								
Indoor	Living room	50	47.5	11.5	49.0	27.0	73.0	0.38
	Bedroom	50	48.3	11.5	49.0	26.0	80.0	
	Average	50	47.9	11.0	48.8	26.5	76.5	** < 0.05**
Outdoor		42	67.3	18.1	72.4	13.0	94.5	

Significant values are in [bold].

**Figure 3:**
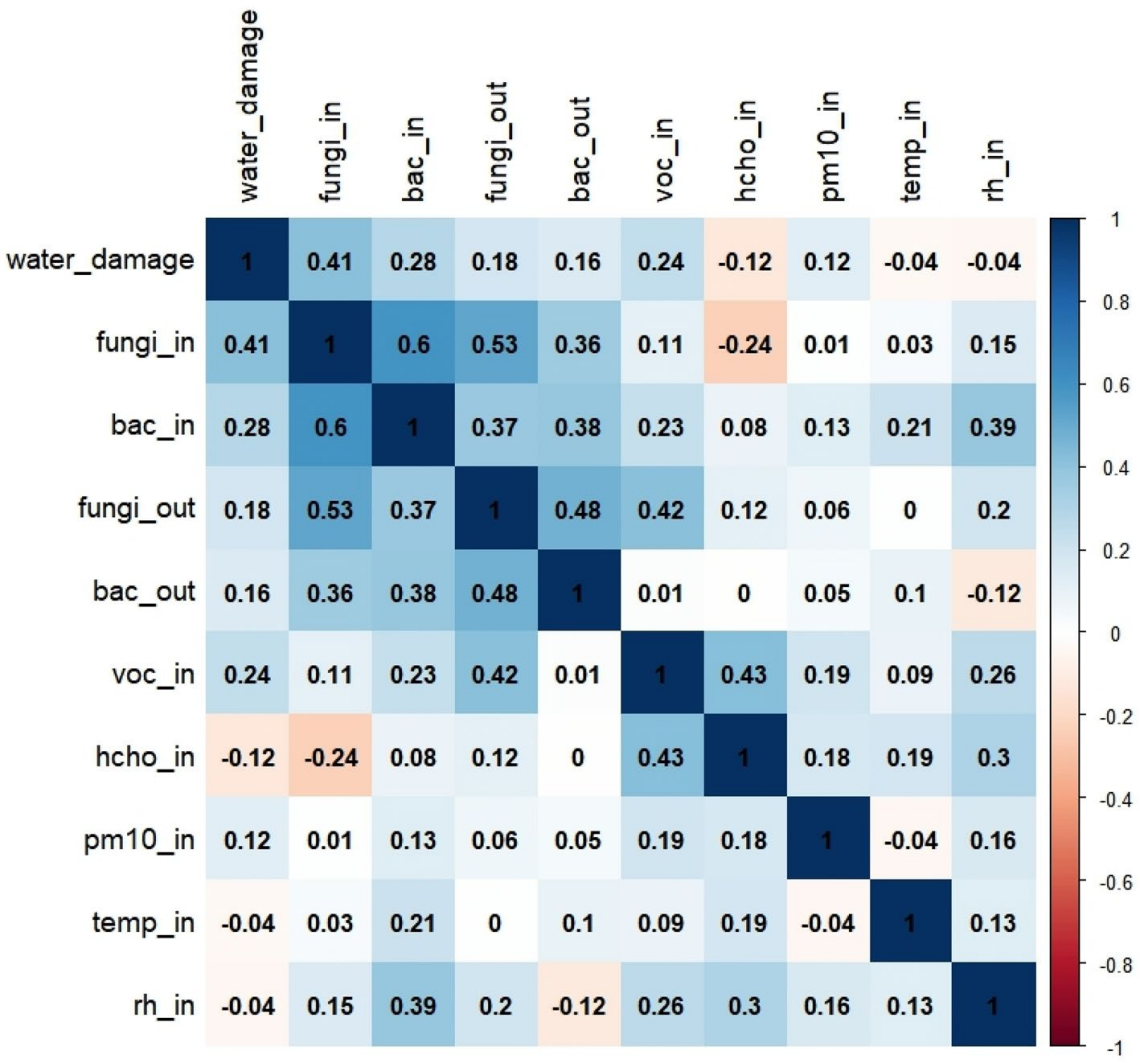
Simple correlation matrix between water damage determined by IR thermal camera and the concentration levels of airborne molds, bacteria, TVOC, formaldehyde, PM_10_, temperature, and relative humidity in the indoor and outdoor environments. * Abbreviations: fungi_in (indoor mold); bac_in (indoor bacteria); fungi_out (outdoor mold); bac_out (outdoor bacteria); voc_in (indoor volatile organic compounds); hcho_in (indoor formaldehyde); pm10_in (indoor PM_10_), temp_in (indoor temperature); rh_in (indoor relative humidity).

In a simple correlation matrix, water damage was significantly correlated with indoor levels of airborne molds (*r* = 0.41) and bacteria (*r* = 0.28), and the Pearson correlation coefficient between the indoor and outdoor levels of airborne molds was highest (*r* = 0.53) (Fig. 3). In the multiple regression analysis, the indoor mold and bacterial levels were also significantly correlated with several independent variables, such as the presence of water damage determined by IR camera and ISAAC questionnaire survey, building age, net square meter area, and frequent natural ventilation, and the adjusted R-squared values were 0.55 for indoor mold and 0.25 for indoor bacterial levels, respectively (*p* < 0.05). All variance inflation factor (VIF) values were below 10 (Table 4).

**Table 4:** Results of multiple regression analysis for indoor mold and bacteria levels (natural log transformed) with dummy variables (environmental factors).

Variable	Mold (CFU/m^3^)			Bacteria (CFU/m^3^)			VIF
	Estimate (β)	Std. Error	*p*-value	Estimate (β)	Std. Error	*p*-value	
(Intercept)	4.70	0.64	** < 0.001**	4.97	0.57	** < 0.001**	–
Water damage determined by IR camera	0.50	0.21	** < 0.05**	0.14	0.18	0.48	1.45
Water damage, leakage, or mold growth (survey)	− 0.47	0.22	** < 0.05**	0.12	0.19	0.53	1.22
Building age	0.35	0.24	0.15	0.47	0.21	** < 0.05**	1.64
Net square meter area	− 0.43	0.27	0.12	− 0.67	0.24	** < 0.05**	1.71
Type of housing (apartment complex/single house)	0.42	0.28	0.14	0.43	0.25	0.08	1.55
Floor	0.01	0.25	0.97	− 0.05	0.23	0.84	1.19
Direction of housing	− 0.40	0.28	0.15	− 0.24	0.25	0.33	1.22
Secondhand smoke	0.05	0.21	0.80	− 0.03	0.18	0.89	1.54
Frequency of natural ventilation	− 0.60	0.29	** < 0.05**	− 0.04	0.26	0.88	1.24
Frequency of cleaning	− 0.04	0.19	0.84	0.15	0.17	0.38	1.25
Pet	0.32	0.23	0.16	0.38	0.20	0.06	1.28
Adjusted R^2^	0.55		** < 0.05**	0.25		** < 0.05**	–

Significant values are in [bold].

**Figure 4:**
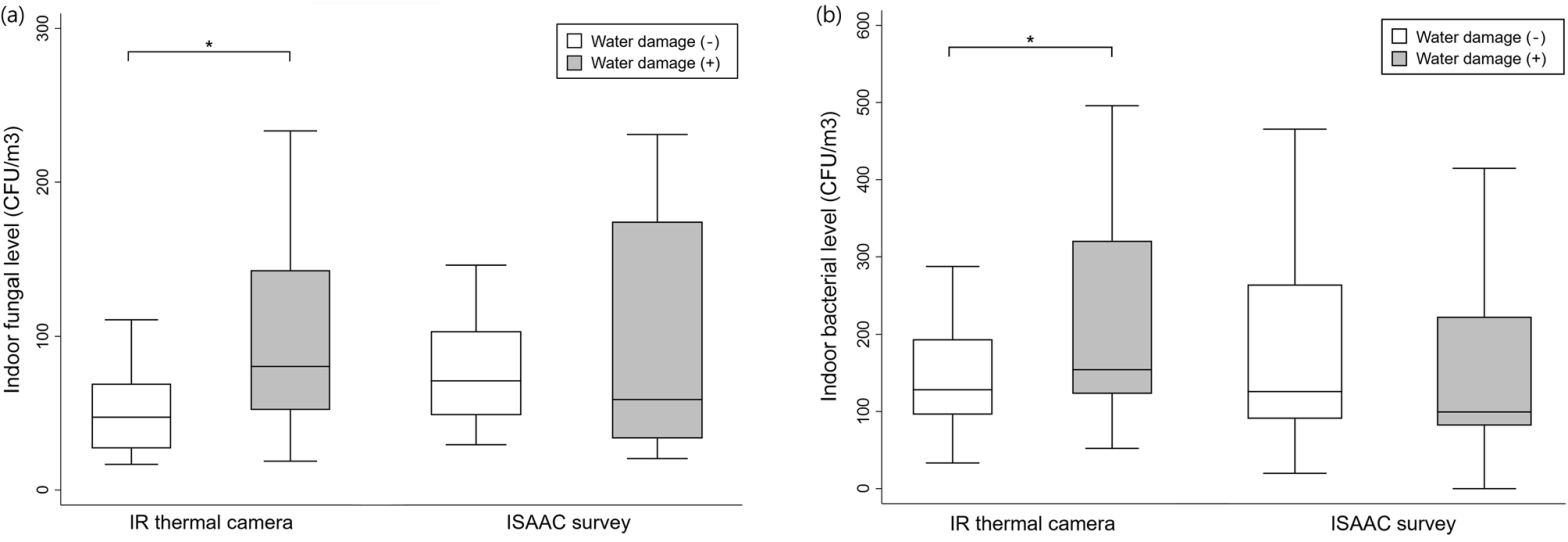
Box plots of (**a**) indoor mold and (**b**) indoor bacterial concentration levels by the presence or absence of water damage using an IR thermal camera and ISAAC questionnaire survey (* *p* < 0.05).

In Fig. 4, box plots of indoor molds and bacterial levels were drawn by the presence of water damage determined by IR thermal imaging camera and questionnaire survey. The mean indoor mold and bacterial levels at water-damaged dwellings (+) were significantly higher than those without water damage (−) when determining water damage based on the photo images taken by IR thermal camera (*p* < 0.05). When using ISAAC questionnaire survey method, on the other hand, the indoor mold and bacterial levels were higher without water damage (−) than with water damage (+), and there was no significant difference.

Unadjusted crude odds ratios (ORs) of asthma, allergic rhinitis, and AD for children in the univariate logistic regression analysis were estimated by environmental and lifestyle factors (Supplementary Table). In Table 5, the crude and adjusted ORs of allergic diseases were estimated by the presence or absence of water damage and high and low levels of indoor mold exposure determined using three different methods. The crude OR of allergic disease was 3.80 (95% CI 1.02–14.21) with positive water damage (+) using the method 1 (IR camera). The adjusted ORs of 3.45 (0.85–13.97) for allergic disease and 1.52 (0.42–5.43) for atopic dermatitis were not statistically significant. Both crude and adjusted ORs of allergic rhinitis were significantly associated with positive water damage (+) using method 1 (crude OR = 9.29, 1.09–78.86; adjusted OR = 10.36, 1.26–119.00) (*p* < 0.05). When using the method 2 (ISAAC questionnaire survey), all crude and adjusted ORs of all allergic diseases were less than 1 with positive water damage, leakage, or mold growth (+), indicating a converse correlation. In the case of using the method 3 (exposure measurement), the adjusted ORs for allergic disease and AD were also elevated with high indoor mold levels but were not statistically significant.

**Table 5:** Adjusted ORs of childhood allergic disease using three different methods of exposure assessment (IR thermal imaging camera, ISAAC questionnaire survey, and environmental measurement sampling).

(a) Allergic disease							
	Variable	Allergic disease (n = 27)		Crude OR	95% CI	Adjusted OR**	95% CI
		Ever diagnosed*	Never diagnosed				
Method 1: Thermal imaging technique-based	Water damage determined by IR camera Positive (≥ 0.2 m^2^) Negative (< 0.2 m^2^)	12 15	4 19	**3.80** –	**1.02–14.21** –	3.45 –	0.85–13.97 –
Method 2: Questionnaire survey-based	Water damage, leakage, or mold growth Positive Negative	17 10	17 6	0.60 –	0.18–2.02 –	0.56 –	0.15–2.08 –
Method 3: Exposure measurement-based	Indoor mold level*** High Low	16 11	9 14	2.26 –	0.73–7.05 –	3.08 –	0.88–10.81 –

(b) Allergic rhinitis							
	Variable	Allergic rhinitis (n = 36)		Crude OR	95% CI	Adjusted OR**	95% CI
		Ever diagnosed	Never diagnosed				
Method 1: Thermal imaging Technique-based	Water damage determined by IR camera Positive (≥ 0.2 m^2^) Negative (< 0.2 m^2^)	15 21	1 13	**9.29** –	**1.09–78.86** –	**10.36** –	**1.26–119.00** -
Method 2: Questionnaire survey-based	Water damage, leakage, or mold growth Positive Negative	23 13	11 3	0.48 –	0.11–2.05 –	0.70 –	0.13–3.75 -
Method 3: Exposure measurement-based	Indoor mold level*** High Low	17 19	8 6	0.67 –	0.19–2.33 –	0.62 –	0.15–2.60 -

(c) Atopic dermatitis							
	Variable	Atopic dermatitis (n = 29)		Crude OR	95% CI	Adjusted OR**	95% CI
		Ever diagnosed	Never diagnosed				
Method 1: Thermal imaging technique-based	Water damage determined by IR camera Positive (≥ 0.2 m^2^) Negative (< 0.2 m^2^)	10 19	6 15	1.32 –	0.39–4.45 –	1.52 –	0.42–5.43 -
Method 2: Questionnaire survey based	Water damage, leakage, or mold growth Positive Negative	19 10	15 6	0.76 –	0.23–2.57 –	0.69 –	0.19–2.52 -
Method 3: Exposure measurement-based	Indoor mold level*** High Low	16 13	9 12	1.64 –	0.53–5.09 –	1.69 –	0.53–5.43 -

*Children who have ever suffered and diagnosed with 2 of 3 allergic diseases (e.g., asthma and atopic dermatitis) are classified into a group of “ever diagnosed”.

**The regression model was adjusted for age, sex, and secondhand smoke and calculated adjusted ORs.

***Indoor mold exposure levels were classified into two groups (high and low) divided by a mean value.

## Discussion

In this cross-sectional study, we found that high levels of indoor microorganisms were related to water damage and leakage (i.e., moisture problems) on the walls and ceilings of residential dwellings, and the inhalation exposure to water damage, hidden dampness, indoor mold was significantly associated with ever-diagnosed allergic diseases among preschool and school children living at the dwellings located in Seoul and the metropolitan area in South Korea. Furthermore, the indoor levels of airborne mold and bacterial concentrations measured in the water-damaged dwellings were significantly higher than those in nondamaged housing. The adjusted OR of allergic rhinitis among the children was significantly elevated by 10.4 times large in the water-damaged dwellings, when determining the water damage and hidden dampness using the IR camera. However, when the presence of water damage, leakage, and visible mold growth was determined and classified using the self- or parent-reported questionnaire survey, all ORs for allergic diseases were below 1, indicating that there might be underestimation or inverse association. Using the questionnaire-based method, the indoor mold and bacterial levels were also higher at nondamaged dwellings than at water-damaged ones. On the other hand, using the exposure sampling approach, the odds of allergic diseases and atopic dermatitis (AD) were also elevated with approximately from 1.7 to 3 times as the levels of indoor mold exposures were high but were not statistically significant. Therefore, our study results showed that there is a positive association between the exposure to hidden molds and dampness at water-damaged dwellings determined by an IR camera and prevalent allergic diseases. That is, the IR thermal technique can detect water damage, hidden dampness, and mold growth more quickly and accurately on site, compared to the other assessment tools. This allows residences to reduce possibility of potential exposure to invisible mold and hidden dampness (moisture) in the indoor environment.

Several studies reported that the IR thermal imaging technique is a more efficient, accurate, reliable, and faster tool for qualitative exposure assessment than other traditional methods when detecting invisible or hidden molds, dampness (moisture), air leakage, and water damage problems generated from internal defects (e.g., cracks, holes, damages, etc.) on the walls, ceilings, and wooden materials of various historic buildings, such as churches, monuments, libraries, residential dwellings, and other old-aged traditional buildings^22–24,34–36^. Furthermore, Seo et al*.* also similarly used an IR thermal camera along with walk-through visual observation to evaluate airborne mold and bacteria levels increased by indoor dampness (moisture) and water damage, and the authors confirmed a positive association between the presence of water damage and the severity of AD in children^27^. In another study conducted by Hwang et al*.* the water damage and leakages at 25 daycare centers in Seoul were determined using an IR thermal camera, and the indoor mold levels were significantly increased at the water-damaged facilities^25^. Similar to the two previous studies, well-trained field inspectors (researchers) visited 50 residences of study participants and used IR thermal imaging cameras to identify water damage, leakage, dampness (moisture), and visible mold growth on the floor, walls, and ceilings in this study. However, due to either false negative (non-detected but water-damaged) or false positive (the presence of water damage detected but not actually moisture or dampness existed), the IR thermal imaging technique still has the possibility of potential misclassification error on the exposure to water damage, dampness, and visible mold growth. To minimize the misclassification of exposure, we performed environmental measurement sampling for TVOC, HCHO, PM_10_, and airborne mold and bacteria, and we found that the levels of airborne mold and bacteria in the water-damaged dwellings were significantly higher than those of non-damaged dwellings, indicating that water damage, dampness and mold growth determined by IR camera were not potentially misclassified but accurately assessed.

Regarding potential misclassification of exposure, however, it has been reported that the self- or parent-responded questionnaire survey approach has limitations in misclassification of exposure due to recall bias. In a longitudinal population-based cohort study, Norbäck et al*.* reported that dampness and mold exposures were related to new onset asthma, but response bias might occur and lead to misclassification of exposures influencing the associations at water-damaged dwellings in Europe^37^. Hahm et al*.* also reported that recall bias occurred because the information on historical exposure to visible molds and dampness and prevalence of allergic rhinitis was retrospectively collected and responded to by school children (12–13 years old) and their parents in a nationwide cross-sectional study^14^. In another cross-sectional study, Lee et al*.* also reported that qualitative and quantitative assessments for environmental risk factors in the questionnaire were lacking; thus, there might be some bias regarding indoor mold exposure, severity and onset age (timing) of childhood asthma^19^. In a review study, Chun et al*.* concluded that it is difficult to identify the associations between genetic, environmental, and socioeconomic factors and the risk of allergic rhinitis using questionnaire surveys; thus, they suggested that a randomized case‒control study should be conducted in the future^38^.

In a longitudinal study conducted by Lee et al*.* the Korean version of the ISAAC questionnaire validated by several previous studies was also used to investigate exposure to various environmental factors, including indoor pollution, in Ulsan city and the prevalent risk of childhood allergic diseases, especially allergic rhinitis and AD, and the authors minimized recall bias by using information on physician-diagnosed or physician-treated allergic diseases^20^. In China, Ellie et al*.* conducted a cross-sectional study and mentioned that there was recall bias generated from parent-reported questionnaire responses and perception differences in their disease symptoms^39^. Another study conducted by Wang et al*.* also used a questionnaire survey developed from the CCHH (China, Children, Homes, Health) project and found strong associations between exposure to dampness and mold and allergic rhinitis and eczema, but their study results were limited due to potential selection and information bias^40^. Despite the bias, most authors explained that the questionnaire used was globally validated and standardized by previous studies, and they emphasized that recall or selection bias was minimized and less likely to occur due to a high response rate of approximately 80%.

In the ISAAC survey, the respondents are required to subjectively response the only visible water damage and presence of mold growth on the wall and ceiling of dwellings that can be seen with the naked eye at certain time point in the past. In other words, the respondents are unable to investigate, find, and assess hidden mold behind walls, ceilings, or other areas in the indoor environments. It is very challenging to visually detect or confirm hidden mold growth and water damage in some areas such as wall cavities^41^. Mold and water damage existing in hidden spaces or areas behind walls can still have a significant impact on indoor air quality, even though they may not be visible. This hidden mold, water damage, and dampness can influence the concentration of mold and bacteria in the indoor air^42^. The evaluation of presence of indoor mold exposure based on respondents' assessments of visible water damage, dampness, and mold growth with their naked eyes may lead potential misclassification. In this regard, we considered the questionnaire method assessing water damage and mold growth throughout visual inspection in the ISAAC survey were quite unreliable. Therefore, there still is high possibility of misclassification in their survey responses, as the results may not accurately reflect the actual indoor mold exposure in the perspective of exposure assessment approach.

Other traditional methods and techniques^4,6,17,43–47^, such as biological air and dust samplings, culture-based analysis, morphology techniques, and microbial volatile organic compound (VOC) measurements^48–51^, were used to evaluate the levels of molds and bacteria measured in indoor and outdoor environments, including residential dwellings (households), daycare centers, kindergartens, schools, and elderly care centers. However, many previous studies reported several limitations. Due to the lack of repeatability in the samples of airborne molds and bacteria collected for a short period of time (within 5 min), it is difficult to determine whether these were representative genera, species or strains^45,52–54^. There are also significant differences in mold concentrations and species collected from the air and dust samples^55^. Nonviable (not growing on the culture media) or nonculturable microorganisms cannot be fully identified and quantified^46,56^. Due to limited distinction of spores collected from local and long distances, the exact source of exposure cannot be found^57^. A variety of environmental conditions, such as temporal or seasonal variation, relative humidity, temperature, air exchange rate, etc., significantly influenced the levels of airborne mold concentrations^6,25,58^. Under- or overestimation in the measurement results might occur due to unstable conditions during air sampling, such as the flow rate and ambient air flow^53^. The collected samples can also be potentially contaminated by samplers (technicians’ contact) or the surrounding environments during the course of aerosol sampling, storage, sample transport, and laboratory analysis^59^. To overcome and fix the weaknesses or limitations in conventional and traditional sampling techniques, new cutting-edge methods and technologies, such as IR thermal imaging cameras with on-site visual inspection^25,27^ and high-throughput DNA/RNA-based sequencing techniques, including 16S sequencing for bacteria, internal transcribed spacer (ITS) region and 18S sequencing for molds, with real-time quantitative polymerase chain reaction (RT‒PCR)^60–63^, should be used and applied for more reliable and accurate results in future studies.

Similar to our study results, several previous studies reported adverse health effects on childhood respiratory, allergic, and skin diseases and related symptoms due to indoor microbial and dampness (moisture) exposures. In Korea, indoor mold and water leaking significantly increased the prevalent risks of allergic diseases (asthma, allergic rhinitis, AD) in low-income households^64^. Yang et al*.* also reported that combined antibiotic use and mold exposure in infancy increased the risk of allergic rhinitis by approximately 1.5-fold^65^. In another study conducted by Hahm et al*.* visible mold or dampness exposure increased the risks of allergic rhinitis (OR = 1.28) and rhinoconjunctivitis (OR = 1.23) in middle-age children (12–13 years old), and total IgE levels ≥ 78 kU/l (i.e., positive) also increased the risks of allergic rhinitis (OR = 1.44) and rhinoconjunctivitis (OR = 1.99)^14^. Similarly, Lee et al*.* reported that indoor mold exposure during the prenatal period was significantly associated with AD (OR = 1.36) via IgE-mediated allergic inflammation in a prospective population-based birth cohort study^66^. Yoon et al*.* also reported that asthmatic children with high levels of indoor mold and positive mold allergens, such as *Alternaria sp., Aspergillus sp., and Penicillium sp.*, significantly increased the severity of bronchial hyperresponsiveness, which might cause chronic allergic and respiratory diseases and related symptoms^67^. On Jeju Island, the mean level of indoor mold at residential houses with school children diagnosed with AD was 59.4 ± 26.6 CFU/m^3^, higher than that at homes for children without AD, 45.5 ± 20.1 CFU/m^3^^68^.

In the U.S., Sharpe et al*.* performed questionnaire surveys for children, adolescents, and adults participating in the NHANES 2005–2006 study, and the authors found that mildew/musty odor exposure significantly increased childhood asthma (OR = 1.92), adult eczema (OR = 1.92) and asthma (OR = 1.61)^52^. In China, Deng et al*.* conducted a large-scale nationwide cross-sectional study and observed that indoor renovation and exposure to mold and dampness significantly increased the risk of allergic diseases and symptoms, especially eczema (OR = 1.40) during the prenatal period and asthma (OR = 1.80) during the postnatal period. More importantly, current childhood allergic symptoms were strongly related to mold and dampness exposure in indoor homes^69^. In another large cross-sectional study conducted by Wang et al. indoor mold spots and damp stains significantly increased asthma 2.5-fold, allergic rhinitis 1.8-fold, and eczema 1.5-fold, and the presence of water damage at current dwellings within the past 12 months also increased the risks of allergic diseases among young parents, asthma 1.8-fold, allergic rhinitis 1.5-fold, and eczema 1.4-fold^40^.

In Europe, a joint birth cohort prospective study was conducted, and the authors found that higher richness in bacteria significantly decreased the risk of childhood allergic rhinitis by approximately 80%. However, high bacterial and fungal diversity was significantly associated with an increased risk of inhalant atopy^62^. In another European longitudinal cohort study conducted by Norbäck et al. indoor mold and dampness significantly increased the incident risk of asthma in adults (OR = 1.49), and approximately 5–15% of onset asthma was attributed to dampness exposure at water-damaged dwellings^37^. In the Northern Europe (RHINE) study, Wang et al*.* found that dampness/mold exposure significantly increased risk of asthma (OR = 1.33–1.62), and AD was also significantly associated with visible mold (OR = 1.35) and dampness/mold (OR = 1.18–1.38). In particular, they observed that there were stronger health effects due to the prolonged dampness/mold exposure at homes^70^. Therefore, many domestic and international studies have shown consistency in the associations between indoor mold and dampness exposure and childhood allergic diseases and related symptoms, and the indoor mold levels in water-damaged homes were generally higher than those in nondamaged homes, similar to our study findings.

According to recent studies^71–76^, the residential buildings with the presence of water damage and hidden dampness showed significantly higher levels of indoor molds and bacterial concentrations. Similarly, on-site exposure assessments were conducted using the IR thermal camera to determine the presence of water damage and hidden moisture. In the housings with indoor water damage, the indoor levels of molds and bacteria were significantly higher than those in homes without water damage. Therefore, the IR thermal camera has the potential to be a useful screening tool for evaluating indoor molds and moisture exposures in dwellings more easily and efficiently by evaluating the water damage on-site, rather than directly measuring the indoor mold and bacterial levels. Similar to previous studies^77–81^, exposure to water damage, molds, and dampness in indoor environments (e.g., buildings, housings, etc.) has been also observed to a significant association with allergic diseases (i.e., rhinitis, asthma, etc.). These findings align with our study results.

Moreover, a study reported that, despite different sources of origin, a correlation between molds and bacteria grown in various building materials was observed^82^. In general, microbial growth is significantly related to environmental factors such as temperature, humidity, and nutrients^83^. Temperature and humidity, in particular, play crucial roles in the growth of both molds and bacteria, even though their sources of occurrence may differ. Therefore, environments characterized by high temperatures and humidity levels tend to exhibit relatively higher growth of molds and bacteria, showing a correlated pattern^84^. However, it's worth noting that molds are more influenced by humidity, whereas bacteria are more affected by temperature^85^. In our current study, we similarly observed a high correlation between indoor molds and bacterial levels during the hot and humid environmental conditions of the summer and early fall seasons in South Korea. This suggests that the indoor environment during these seasons, characterized by high temperatures and humidity, may facilitate the growth of both molds and bacteria, leading to the high correlation observed in this study.

However, there are some limitations in the present study. First, this is a cross-sectional study design, and the total number of study subjects (i.e., sample size) was relatively small (n = 50). Similar to our study, however, several recent studies showed statistically significant results, although the number of study participants and environmental samples collected was also small (n < 100)^17,25,46,49,68,86,87^. We were also unable to collect the long-term data and information on the exposure to microorganisms, environmental pollutants, environmental conditions, water damage and dampness, mold growth, lifestyle factors, and other biological agents, thus not considering variations, trends or differences in seasons, other geographical locations, indoor and outdoor residential buildings, sampling and analytical devices, and socioeconomic status. In future studies, a larger sample size for the study subjects and repeated measurements during the long-term period must be collected to increase reliability, validity, and reproducibility in the results. Second, indoor microbial samples were collected not by personal but by fixed-location monitoring; thus, individual time-activity patterns at each microenvironment were not fully considered, which might lead to underestimation of the exposure levels. In fact, social and technological restrictions (limitations) always exist in every study, in which children and their parents are included as the study participants because they cannot wear heavy personal monitors for safety & health or other kinds of issues in real-world situations. In this regard, we collected all the samples at the center of each location, where the study subjects use the most frequently and spent for the longest time period; thus, the possibility of exposure misclassification in their indoor environments was fully controlled and increased the representativeness of our sampling results.

Finally, characterization of mold and bacterial genera, species and strains related to the prevalence of allergic diseases was not performed. However, we are able to anticipate which genera or species of molds and bacteria are dominant based on the results of previous studies. The arithmetic mean (AM) of indoor molds collected at various multiuse public facilities (e.g., kindergartens, hospitals, elderly care centers, etc.) in the autumn season in Korea was 368.8 CFU/m^3^, and 10 genera of molds, such as *Penicillium sp., Cladosporium sp., Alternaria sp., and Aspergillus sp.,* were dominant. In another study, 16 genera of molds, such as *Penicillium sp., Aspergillus sp., Cladosporium sp., Alternaria sp., *etc*.*, were also characterized using PCR purification kits at the water-damaged dwellings, and *Cladosporium sp.*, in particular, was mainly dominant in the autumn and winter seasons^16^, similarly observed in another recent study^88^. Kim et al*.* also found that the mean levels (GMs) of indoor bacteria were 417.3 CFU/m^3^ in the bathroom, 324.9 CFU/m^3^ in the kitchen, 275.7 CFU/m^3^ in the living room, respectively, and gram-positive bacteria, such as *Staphylococcus sp.* (19%), *Micrococcus sp.* (16%), and *Bacillus sp.* (11%), and gram-negative bacteria, such as *Pseudomonas sp.* (4%), *Acinetobacter sp.* (3%), etc., were observed at residential dwellings in Seoul and the metropolitan area in Korea^17^. In future studies, species, strains, or components of airborne molds and bacteria collected from water-damaged dwellings should be identified and characterized using the high-throughput DNA/RNA-based sequencing techniques.

Despite the limitations mentioned above, the present study has several strengths. First, to the best of our knowledge, this is the first study to compare three different methods of exposure assessment for classification of indoor mold exposure and the presence of water damage and hidden dampness in Korean residential housings. In our results, we found that the IR thermal imaging camera can be used as a good screening tool to detect hidden dampness and water damage more quickly and accurately than the other methods. Our study results are also reliable because field inspectors not only identified water-damaged areas on the walls and ceilings of housing by taking photos using the IR thermal camera but also investigated other environmental conditions, including indoor and outdoor temperature, relative humidity, natural ventilation, hidden dampness (moisture), water stains, visible mold, and moly odor, via field inspection (visual observation) on site. In a review study, indoor residential dampness or mold (D/M) in homes identified by visual or smell observation-based assessment (field inspection) without microbiological cultivation and measurements was associated with asthma onset, wheezing, respiratory infections, and allergic rhinitis among children^89^. In the same manner, we also performed visual and olfactory evaluation to determine the presence of water damage, leakage, mold growth, and moldy odor in dwellings and cross-checked their observations with photo images taken by the IR camera in our study.

Furthermore, using all available tools, including the globally validated and standardized ISAAC questionnaire, we collected the detailed information on demographic characteristics, housing type, environmental and lifestyle factors (qualitative) and environmental measurement data of airborne microorganisms and environmental pollutants (e.g., TVOCs, HCHO, PM_10_) (quantitative). Although the sample size was relatively small (n ≤ 50), we provided evidence that indoor mold exposure and water damage (dampness) determined by the IR camera were used to evaluate the associations with prevalent allergic disease in children based on the qualitative information and quantitative exposure data collected from the present study. We also performed sensitivity analysis to support evidence on the reliability of the IR thermal camera. The results showed sensitivity 45%, specificity 83%, and accuracy 62% for prevalent allergic diseases by the IR thermal camera; sensitivity 63%, specificity 26%, and accuracy 46% by the ISAAC questionnaire survey; and sensitivity 59%, specificity 60%, and accuracy 60% by environmental sampling, respectively. The IR thermal camera showed the highest specificity (83%) and accuracy (62%) among the three assessment tools. The positive predictive values were also calculated as 75% for IR camera, 64% for environmental sampling, and 50% for ISAAC questionnaire survey.

We also measured and characterized both indoor microbial (molds and bacteria) and environmental pollutant (TVOCs, HCHO, and PM_10_) levels at the dwellings, and the levels of airborne molds and bacteria were less than the legal exposure limit values, 500 CFU/m^3^ for molds and 800 CFU/m^3^ for bacteria. We also found that high levels of indoor microbial concentrations at the dwellings were correlated with the presence of water damage and hidden dampness (moisture) determined using the IR thermal camera, similar to two previous studies^25,27^. In the present study, however, the association between indoor mold and water damage exposure at dwellings and prevalent atopic dermatitis (AD) was different and inconsistent from the results of previous studies. This might come from some differences in study design, characteristics of study participants (e.g., age, sex, children’s health condition, medical history, socioeconomic factors, etc.), clinical evaluation method (Doctor-evaluated SCORAD vs. Parents-reported medical history using ISAAC questionnaire), period of data collection (environmental monitoring, on-site inspection, and questionnaire survey), and geographic location of target subject dwellings.

In addition, the allergic (or atopic) march could be considered for these different results. The development of AD in infancy and subsequent allergic in later childhood is known as the allergic march^90^. The doctor-diagnosed patients with AD aged 3–4 years in the previous study have moderate or severe symptoms. For this reason, the significant association of exposure to indoor mold or water damage with prevalence of AD was observed. In this study, however, the onset or development of allergic rhinitis and asthma may be observed in atopic dermatitis patients aged 9 or older from the perspective of the allergic march. We believe that the observation of a significant association between allergic rhinitis and the degree of water damage would support for this explanation. The ISAAC questionnaire methodology does not provide detailed information on the patient’s severity. The weak or little association of the prevalence of AD with mold/water damage exposure may also be attributed to the limitation of this survey characteristics. Rather, this discussion may not be consistent with other situations. If possible, a new pooled analysis combining with all data and information collected from the two previous and present studies, should be carried out to increase the sample size, identify the exact association between exposure and disease, and guarantee higher reliability in the study results.

Therefore, further studies should be conducted in the future. First, the indoor mold species, components, and strains most dominant in Korean residential housings (single-stage and multicomplex types) and associated with allergic diseases should be characterized. Second, it is also necessary to identify which environmental factor and what reason influenced the growth of these indoor microorganisms, for instance, poor ventilation due to building structure problems, cracks or defects outside buildings, contaminated building materials, home interior renovations, inflow of outdoor air pollution, resident lifestyles, etc. More detailed information on confounding factors, including genetic and socioeconomic factors, related to the prevalent risk of childhood allergic diseases must be also collected to identify a causal relationship between the exposure and disease (severity of symptoms) in the data analysis. In the future, further epidemiological studies, such as a population-based longitudinal study^54^, a nested case‒control study^91^, a randomized controlled intervention trial study^92,93^, and a repeated cross-sectional study^94^, would be necessary.

In conclusion, this study provided evidence that high levels of indoor mold exposure due to the presence of water damage and hidden dampness in 50 household dwellings in Korea were significantly associated with childhood allergic rhinitis. Using three different assessment tools, we determined the presence of water damage, leakage, fungal growth, and hidden dampness (moisture problems) and accurately classified the environmental exposures, and this will provide the opportunity to develop appropriate countermeasures and take actions to reduce and control environmental exposure to indoor molds and dampness (moisture) at water-damaged dwellings. In the future, a large-scale longitudinal study should be conducted to characterize the genera, species, and strains of molds, to identify the main reasons for the growth of indoor microorganisms in water-damaged dwellings, and to suggest an accurate, efficient, reliable, and validated assessment tool for identification of the associations between exposure to environmental, biological, genetic, and socioeconomic factors and prevalent risk of childhood allergic diseases.

### Supplementary Information

Supplementary Table 1.

## Data Availability

The informed consent given by this study participants does not cover data sharing or posting in publicly accessible databases. However, the data will be available upon request by means of a project agreement from the authors. Requests should be sent to the corresponding author and are subject to approval by all named authors participating in this study.
