# Supplementary material for: Association of exposure to indoor molds and dampness with allergic diseases at water-damaged dwellings in Korea
Source: Sci Rep. 2024 Jan 2;14:135. doi: 10.1038/s41598-023-50226-w (PMC10762174; doi:10.1038/s41598-023-50226-w)
Supplement: Supplementary file 1 — Supplementary Table 1. [file 41598_2023_50226_MOESM1_ESM.docx]

**Supplementary Table**. Unadjusted ORs of allergic disease (asthma, allergic rhinitis, and atopic dermatitis) for children by environmental and lifestyle factors.

* Children who have ever suffered from 2 of 3 allergic diseases (e.g., asthma and atopic dermatitis) are classified into a group of “ever diagnosed”; ** Unit: US dollar per month ($1=\1,308.9 Korean won); *** Actual occupation area of residence

| Variable | Classification | Allergic disease (n=27)* | | | Asthma (n=8) | | | Allergic rhinitis (n=36) | | | Atopic dermatitis (n=29) | | |
| --- | --- | --- | --- | --- | --- | --- | --- | --- | --- | --- | --- | --- | --- |
|  |  | Crude  OR | 95% CI | p-value | Crude  OR | 95% CI | p-value | Crude  OR | 95% CI | p-value | Crude  OR | 95% CI | p-value |
| Age | <10 years-old | 1.00 | - | - | 1.00 | - | - | 1.00 | - | - | 1.00 | - | - |
|  | ≥10 years-old | 1.33 | 0.43-4.09 | 0.62 | 0.73 | 0.15-3.44 | 0.69 | 4.10 | 0.98-17.20 | 0.05 | 0.40 | 0.12-1.25 | 0.12 |
| Sex | Male | 1.00 | - | - | 1.00 | - | - | 1.00 | - | - | 1.00 | - | - |
|  | Female | 2.18 | 0.68-6.96 | 0.19 | 0.19 | 0.02-1.69 | 0.14 | 0.33 | 0.09-1.18 | 0.09 | 1.41 | 0.44-4.55 | 0.56 |
| Income** | <2,292 | 1.00 | - | - | 1.00 | - | - | 1.00 | - | - | 1.00 | - | - |
|  | 2,292-3,820 | 0.69 | 0.18-2.73 | 0.60 | 0.19 | 0.02-1.90 | 0.16 | 1.08 | 0.22-5.33 | 0.92 | 0.50 | 0.11-2.12 | 0.34 |
|  | ≥3,820 | 1.11 | 0.28-4.42 | 0.88 | 0.64 | 0.12-3.46 | 0.61 | 0.61 | 0.14-2.76 | 0.52 | 0.52 | 0.13-2.15 | 0.37 |
| Type of  housing | Apartment  complex | 1.00 | - | - | 1.00 | - | - | 1.00 | - | - | 1.00 | - | - |
|  | Single/multi-  household | 2.00 | 0.51-7.78 | 0.32 | - | - | - | 0.43 | 0.11-1.71 | 0.23 | 5.00 | 0.97-25.93 | 0.06 |
| Building age | <10 years | 1.00 | - | - | 1.00 | - | - | 1.00 | - | - | 1.00 | - | - |
|  | ≥10 years | 1.04 | 0.31-3.50 | 0.95 | - | - | - | 0.91 | 0.23-3.54 | 0.89 | 1.93 | 0.57-6.58 | 0.29 |
| Duration of residency | <3 years | 1.00 | - | - | 1.00 | - | - | 1.00 | - | - | 1.00 | - | - |
|  | ≥3 years | 0.94 | 0.30-2.91 | 0.91 | 1.13 | 0.24-5.38 | 0.88 | 0.50 | 0.13-1.90 | 0.31 | 1.23 | 0.39-3.85 | 0.73 |
| Net square  meter area*** | ≥85m^2^ | 1.00 | - | - | 1.00 | - | - | 1.00 | - | - | 1.00 | - | - |
|  | <85m^2^ | 0.97 | 0.25-3.73 | 0.97 | 0.39 | 0.08-1.99 | 0.26 | 0.96 | 0.21-4.27 | 0.95 | 1.92 | 0.50-7.41 | 0.34 |
| Direction of housing | South | 1.00 | - | - | 1.00 | - | - | 1.00 | - | - | 1.00 | - | - |
|  | East or West | 0.29 | 0.05-1.65 | 0.16 | 2.47 | 0.39-15.73 | 0.34 | 0.56 | 0.09-2.38 | 0.35 | 0.24 | 0.04-1.37 | 0.11 |
| Floor | ≥6th | 1.00 | - | - | 1.00 | - | - | 1.00 | - | - | 1.00 | - | - |
|  | <6th | 1.89 | 0.61-5.83 | 0.27 | 0.91 | 0.20-4.13 | 0.90 | 0.50 | 0.14-1.78 | 0.28 | **3.80** | **1.16-12.46** | **<0.05** |
| Secondhand smoke | No | 1.00 | - | - | 1.00 | - | - | 1.00 | - | - | 1.00 | - | - |
|  | Yes | 1.59 | 0.52-4.87 | 0.42 | 0.66 | 0.14-3.12 | 0.60 | 0.35 | 0.10-1.27 | 0.11 | 1.74 | 0.56-5.46 | 0.34 |
| Frequency of natural  ventilation | Everyday | 1.00 | - | - | 1.00 | - | - | 1.00 | - | - | 1.00 | - | - |
|  | 2-3times/week | 1.31 | 0.20-8.62 | 0.77 | 1.36 | 0.13-14.02 | 0.80 | 1.63 | 0.17-15.95 | 0.68 | 1.10 | 0.17-7.22 | 0.92 |
| Frequency of cleaning | Everyday | 1.00 | - | - | 1.00 | - | - | 1.00 | - | - | 1.00 | - | - |
|  | 2-3times/week | 0.46 | 0.14-1.50 | 0.20 | - | - | - | **0.16** | **0.04-0.61** | **<0.05** | 0.73 | 0.23-2.38 | 0.60 |
| Pet | No | 1.00 | - | - | 1.00 | - | - | 1.00 | - | - | 1.00 | - | - |
|  | Yes | 0.23 | 0.05-1.05 | 0.06 | 0.46 | 0.05-4.18 | 0.49 | 0.36 | 0.09-1.46 | 0.15 | 0.52 | 0.14-2.01 | 0.34 |
| Mold  (CFU/m^3^) | Quartile 1 | 1.00 | - | - | 1.00 | - | - | 1.00 | - | - | 1.00 | - | - |
|  | Quartile 2 | 0.80 | 0.16-4.12 | 0.79 | 1.10 | 0.13-9.34 | 0.93 | 0.90 | 0.14-5.65 | 0.91 | 1.17 | 0.24-5.64 | 0.84 |
|  | Quartile 3 | 4.80 | 0.86-26.79 | 0.07 | 0.50 | 0.04-6.35 | 0.59 | 0.42 | 0.08-2.36 | 0.33 | 1.19 | 0.23-5.67 | 0.86 |
|  | Quartile 4 | 3.60 | 0.71-18.25 | 0.12 | 1.65 | 0.23-11.99 | 0.62 | 1.00 | 0.16-6.20 | 0.99 | **6.42** | **1.00-41.21** | **<0.05** |
| Bacteria  (CFU/m^3^) | Quartile 1 | 1.00 | - | - | 1.00 | - | - | 1.00 | - | - | 1.00 | - | - |
|  | Quartile 2 | 4.80 | 0.86-26.79 | 0.07 | - | - | - | 2.75 | 0.40-18.88 | 0.30 | 2.33 | 0.46-11.81 | 0.31 |
|  | Quartile 3 | **15.01** | **2.02-111.17** | **<0.05** | - | - | - | 2.50 | 0.36-17.32 | 0.35 | **6.00** | **1.02-35.37** | **<0.05** |
|  | Quartile 4 | 2.57 | 0.47-14.10 | 0.28 | - | - | - | 0.58 | 0.12-2.95 | 0.52 | 4.50 | 0.84-24.18 | 0.08 |
| TVOC  (㎍/m^3^) | Quartile 1 | 1.00 | - | - | 1.00 | - | - | 1.00 | - | - | 1.00 | - | - |
|  | Quartile 2 | 0.21 | 0.04-1.17 | 0.07 | - | - | - | 0.60 | 0.10-3.50 | 0.57 | 0.17 | 0.03-1.11 | 0.06 |
|  | Quartile 3 | 1.88 | 0.34-10.46 | 0.47 | - | - | - | 0.90 | 0.14-5.65 | 0.91 | 4.29 | 0.66-28.79 | 0.13 |
|  | Quartile 4 | 0.73 | 0.15-3.47 | 0.69 | - | - | - | 0.68 | 0.12-3.87 | 0.66 | 2.86 | 0.53-15.47 | 0.22 |
| HCHO  (㎍/m^3^) | Quartile 1 | 1.00 | - | - | 1.00 | - | - | 1.00 | - | - | 1.00 | - | - |
|  | Quartile 2 | 0.45 | 0.09-2.28 | 0.33 | 1.09 | 0.06-19.63 | 0.95 | 2.22 | 0.33-15.18 | 0.42 | 0.61 | 0.13-2.98 | 0.54 |
|  | Quartile 3 | 0.22 | 0.04-1.19 | 0.08 | 4.00 | 0.36-45.10 | 0.26 | 0.62 | 0.12-3.22 | 0.57 | 1.71 | 0.34-8.68 | 0.52 |
|  | Quartile 4 | 0.71 | 0.14-3.61 | 0.68 | 3.60 | 0.32-40.23 | 0.30 | 1.48 | 0.26-8.50 | 0.66 | 1.93 | 0.39-9.60 | 0.42 |
